# Safer Opioid Supply, Subsequent Drug Decriminalization, and Opioid Overdoses

**DOI:** 10.1001/jamahealthforum.2025.0101

**Published:** 2025-03-21

**Authors:** Hai V. Nguyen, Shweta Mital, Shawn Bugden, Emma E. McGinty

**Affiliations:** 1 School of Pharmacy, Memorial University, St John’s, Newfoundland and Labrador, Canada; 2 College of Pharmacy, University of Manitoba, Winnipeg, Canada; 3 Weill Cornell Medicine, New York, New York

## Abstract

**Importance:**

British Columbia, Canada, was the first and only jurisdiction globally to implement a province-wide safer supply policy, which offered pharmaceutical grade opioids to individuals at risk of opioid overdose, followed by the decriminalization of drug possession. Supporters of the safer supply policy argue that this policy could save lives by offering pharmaceutical-grade opioids to people who use toxic street drugs. Similarly, proponents of decriminalization suggest that decriminalizing drug possession could reduce drug overdoses by reducing stigma associated with drug use and enabling persons who use drugs to seek addictions treatment. However, critics of both policies believe that providing safer opioids and removing penalties for drug possession may worsen the crisis. Currently, there is limited evidence on the health impacts of these policies.

**Objective:**

To assess the association of British Columbia’s adoption of the safer supply policy and subsequent decriminalization of drug possession with opioid overdose hospitalizations and deaths.

**Design, Setting, and Participants:**

This observational cohort study used synthetic difference-in-differences analysis with quarterly province-level data to compare prepolicy and postpolicy changes in British Columbia with those in other Canadian provinces that did not implement these policies. The study period spanned from quarter 1 of 2016 to quarter 4 of 2023.

**Exposure:**

Safer opioid supply policy implemented in March 2020 and decriminalization of drug possession implemented in January 2023.

**Main Outcomes and Measures:**

Opioid-poisoning hospitalizations and apparent opioid-related toxicity deaths, measured as number per 100 000 population.

**Results:**

The safer supply policy alone was associated with an increase of 1.66 opioid hospitalizations per 100 000 population (95% CI, 0.41-2.92; *P* = .009) or 33%. The addition of drug possession decriminalization was associated with a further increase of 1.27 opioid hospitalizations per 100 000 population (95% CI, 0.05-2.50; *P* = .046) for an overall 58% increase compared with the period before the safer supply policy was in effect. There was insufficient evidence to conclusively attribute an increase in opioid overdose deaths to these policy changes.

**Conclusions and Relevance:**

This cohort study found that neither the safer supply policy nor the subsequent decriminalization of drug possession appeared to alleviate the opioid crisis. Instead, both were associated with an increase in opioid overdose hospitalizations. The observed increase in opioid hospitalizations, without a corresponding increase in opioid deaths, may reflect greater willingness to seek medical assistance because decriminalization could reduce the stigma associated with drug use. However, it is also possible that reduced stigma and removal of criminal penalties facilitated the diversion of safer opioids, contributing to increased hospitalizations.

## Key Points

**Question:**

What is the association of the adoption of the safer supply policy and subsequent decriminalization of drug possession in British Columbia, Canada, with opioid overdose outcomes?

**Findings:**

Using the synthetic difference-in-differences method, this cohort study found that the safer opioid policy was associated with a statistically significant increase in opioid overdose hospitalizations but no change in overdose deaths; the addition of drug possession decriminalization was associated with a further increase in overdose hospitalizations.

**Meaning:**

Neither the safer opioid supply policy nor the decriminalization of drug possession appeared to mitigate the opioid crisis, and both were associated with an increase in opioid overdose hospitalizations.

## Teaser

This cohort study examined changes in opioid hospitalizations and deaths associated with adoption of first, the safer supply policy and then, the decriminalization policy in British Columbia relative to control provinces in Canada.

## Introduction

Between 2016 and 2020, the number of opioid overdose deaths across Canada more than doubled from nearly 2800 to 6400 deaths, prompting policy measures to curb this crisis.^1^ In March 2020, British Columbia, Canada, implemented the safer supply policy, which allows physician and nurse practitioners to prescribe pharmaceutical-grade opioids (such as hydromorphone and 12-hour sustained-release oral morphine) to those at risk of overdose.^2^ Up to 14 hydromorphone tablets and two 80- to 240-mg oral morphine capsules could be prescribed daily, with requirements for witnessed ingestion determined at the discretion of the prescriber.^2^ The goal of this policy is to protect people who use substances from the unregulated drug supply that is known to be responsible for most overdose deaths.^2^ Subsequently, in January 2023, British Columbia decriminalized possession of small amounts of drugs for personal use among adults. This measure aimed to reduce the stigma associated with drug use, enabling persons who use drugs to seek addictions treatment and lowering costs for the criminal justice system.^3^ Individuals found to possess up to 2.5 g of these substances (select opioids, crack and powder cocaine, methamphetamine, or 4-methylenedioxymethamphetamine [MDMA]) would not face prosecution and instead would be offered access to recovery supports.^3^ Notably, drug possession offenses in 2023 were nearly one-fifth of those in 2019.^4^

As drug overdose deaths in British Columbia continue to rise, the effectiveness of these policies have been widely debated. Critics of the safer supply policy point to evidence suggesting that the prescribed safer supply may be being diverted to the illegal market.^5^ Meanwhile, critics of decriminalization believe that removing penalties for drug possession without addressing the unregulated supply or reducing wait times for addiction treatment will only further worsen the crisis.^6^ In addition, there is frustration over use of drugs in public, which recently prompted the provincial government to recriminalize public use of drugs, although private possession remains decriminalized.^7^ It is also unclear how the decriminalization policy works in the presence of a safer supply policy. On one hand, if people who use substances switch from unregulated opioids to safer supply opioids and decriminalization reduces stigma (as policymakers intend), the 2 policies could together improve overdose outcomes. On the other hand, decriminalization may facilitate the diversion of safer supply opioids, as the diverted supply of small amounts would be exempt from criminal charges.

Two studies have examined the impacts of British Columbia’s safer supply policy alone. Comparing recipients of prescribed safer supply who had opioid use disorder to a matched control group, Slaunwhite et al^8^ concluded that the policy was associated with reduced risk of overdose and all-cause mortality among these individuals. Meanwhile, Nguyen et al^9^ evaluated the population-level effects by comparing changes in opioid overdose hospitalizations and deaths before and after the policy was implemented in British Columbia with corresponding changes in provinces that did not implement this policy. They found that the policy was associated with a statistically significant increase in overdose hospitalizations in British Columbia and no change in overdose deaths.^9^ However, these studies could examine only the short-term effects (up to 2 years) of the safer supply policy. Moreover, the postpolicy period in these studies coincided with the COVID-19 pandemic making it difficult to rule out potential confounding effects of the pandemic.

Although a detailed evaluation of British Columbia’s decriminalization policy is planned,^10^ no findings have been reported to date. Evidence on the effects of decriminalizing drug possession in other jurisdictions is mixed. A few studies have evaluated decriminalization of drugs in US states. Spencer^11^ used the synthetic control method and found that decriminalization was associated with a 23% increase in unintentional drug overdose deaths in Oregon. Joshi et al^12^ used a similar analytical approach but more granular data and found no change in both intentional and unintentional drug overdose deaths in both Oregon and Washington. Using longer postpolicy data and explicitly accounting for the heterogeneous spread of fentanyl across states, Zoorob et al^13^ too noted no association of Oregon’s decriminalization policy with intentional and unintentional drug overdose mortality. Meanwhile, Felix^14^ showed that decriminalization of drugs in Portugal not only reduced drug offenses but was also associated with fewer drug overdose deaths. Apart from being inconclusive, these findings may not be applicable to the unique context of British Columbia where the safer supply and decriminalization policies are concurrently in effect.

Using province-level administrative data and quasi-experimental difference-in-differences design, this study provides the first evidence, to our knowledge, on the association of British Columbia’s decriminalization of drug possession that followed the safer supply policy with overdose outcomes. Using data for a longer time period, it also sheds light on the longer-term impacts of the safer supply policy than was previously studied in Nguyen et al.^9^

## Methods

### Study Period

The study period spanned from quarter 1 of 2016 to quarter 4 of 2023 and was determined by availability of data on study outcomes. The prepolicy period spanned from quarter 1 of 2016 to quarter 1 of 2020, the safer supply period from quarter 2 of 2020 to quarter 4 of 2022 and safer supply plus decriminalization period from quarter 1 to quarter 4 of 2023.

### Study Outcomes and Data Sources

We examined rates of opioid overdose poisoning hospitalizations (*opioid hospitalizations*, hereafter), and deaths from apparent opioid toxicity (*opioid deaths*, hereafter). Data on these outcomes were publicly available from the Public Health Agency of Canada.^1^ Opioid hospitalizations were identified using *International Statistical Classification of Diseases and Related Health Problems, Tenth Revision* codes T40.0, T40.1, T40.2, T40.3, T40.4, and T40.6.^1^ Meanwhile, opioid deaths were defined as a death caused by intoxication/toxic effects (poisoning) resulting from substance use, where 1 or more of the substances is an opioid, regardless of how it was obtained (eg, illegally or through personal prescription).^1^ All data were at the province-quarter level and measured per 100 000 population.

Because this study used publicly available, deidentified aggregate province-level data, it did not require ethics approval per the Newfoundland and Labrador Health Research ethics board. Informed consent from participants was also not required. This study followed the Strengthening the Reporting of Observational Studies in Epidemiology (STROBE) reporting guidelines.

### Statistical Analysis

We used the synthetic difference-in-differences (SDID) method^15^—a novel data-driven technique that combines strengths of both traditional difference-in-differences and synthetic control methods,^16,17,18^ and is able to accommodate potential violations of prepolicy parallel trends while generating more precise estimates.^15^ Specifically, it compared changes in outcomes in British Columbia across 3 time periods with the corresponding changes in a synthetic control group of provinces that closely resembled British Columbia. This synthetic control group was created by reweighting the 6 control provinces (Alberta, Saskatchewan, Manitoba, Ontario, New Brunswick, and Nova Scotia) and prepolicy periods to minimize prepolicy differences between the treatment and synthetic control groups in both the outcomes and variables used to predict outcomes. Quebec was excluded due to lack of data on study outcomes, and Newfoundland, Labrador, and Prince Edward Island were excluded due to the small number of observations for these provinces. The 3 time periods were defined as (1) before either safer supply or decriminalization policy was implemented (*prepolicy* period, hereafter); (2) when only the safer supply policy was in effect (*safer supply* period, hereafter); and (3) when both safer supply and decriminalization were in effect (*safer supply plus decriminalization* period, hereafter).

The SDID regressions controlled for time-varying provincial economic (unemployment rate and consumer price index) and demographic (proportion of male individuals and proportion of individuals aged 0-17 years) characteristics, other opioid policies implemented during the study period (namely, changes in naloxone status from schedule 1 to schedule 2 and over-the-counter) across provinces, and province and quarter-year indicators. Because the safer supply period coincided with the COVID-19 pandemic, which was marked by public health restrictions and reduced access to harm reduction services, the regressions also controlled for the Bank of Canada COVID-19 stringency index. This index captured various types and stringency of public health restrictions (eg, school closures, travel restrictions, stay at-home requirements) during the COVID-19 period (January 4, 2020, to July 15, 2022).^19^

Standard errors were based on permutation inference using 100 placebo replications. Differences in coefficient estimates between the safer supply period and the safer supply plus decriminalization period were tested using the z test. All analyses were conducted using the sdid package in Stata statistical software (version 18; StataCorp, LLC). Tests were 2-sided, and a significance level of *P* < .05 was used.

We examined the sensitivity of our results to exclusion of the COVID-19 stringency index, naloxone policies, and economic indicators. We also excluded Ontario and New Brunswick because small-scale pilot safer supply programs were implemented in these provinces. Third, we restricted the study period to quarter 2 of 2020 onward (ie, after the implementation of safer supply policy) to assess changes in outcomes associated with addition of decriminalization while holding safer supply status constant. Finally, to assess the credibility of the parallel trends assumption, we conducted event study analyses that examined differences in outcome evolution in British Columbia and the synthetic control provinces prior to the adoption of safer supply and decriminalization policies.

## Results

### Graphical Analysis

**Figure 1.**
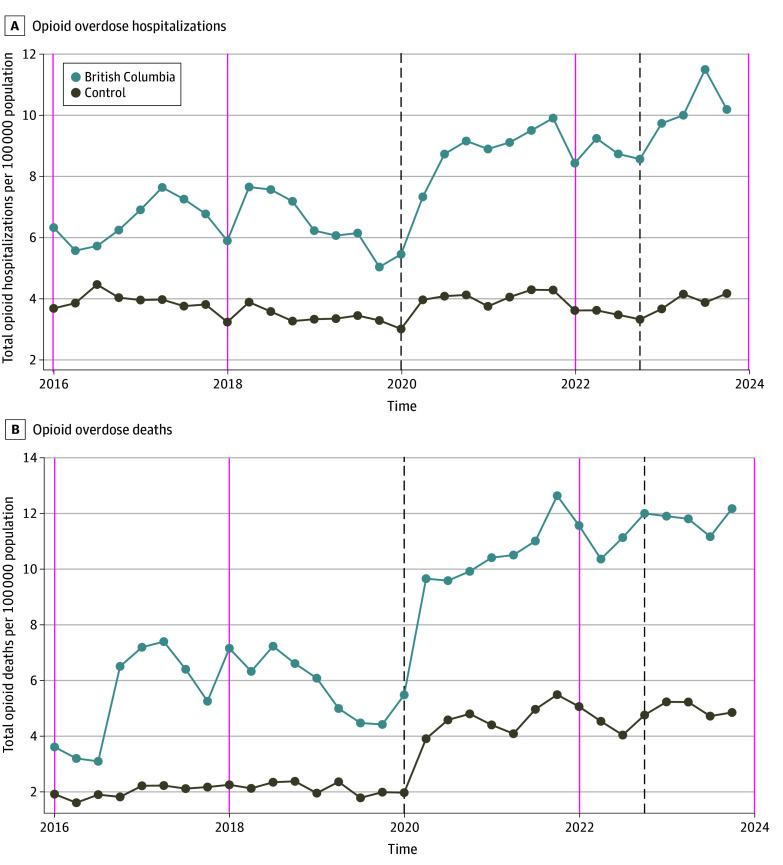
Trends in Outcomes, British Columbia vs Control Provinces Data are from Public Health Agency of Canada for the period from quarter 1 of 2016 to quarter 4 of 2023. Control provinces are Alberta, Saskatchewan, Manitoba, Ontario, New Brunswick, and Nova Scotia. The first vertical dashed line marks implementation of safer supply policy (quarter 1 of 2020 onward) and the second vertical dashed line marks the decriminalization policy (quarter 1 of 2023 onward).

Figure 1 presents trends in the study outcomes in British Columbia and the average control province. There were some fluctuations in British Columbia’s trends from one quarter to the next. For instance, British Columbia experienced a decline in hospitalizations in quarter 1 of 2018, a spike in quarter 2 of 2018, and a subsequent decline until quarter 1 of 2021. Although trends were broadly similar in the control provinces, these fluctuations were much more muted than in British Columbia. After the safer supply policy was implemented, however, there was a sharp spike in opioid hospitalizations (from 5.03 per 100 000 population in quarter 4 of 2019 to 8.73 per 100 000 population in quarter 3 of 2020), which remained around that level until the end of 2022. The opioid hospitalizations further increased sharply after the decriminalization policy was introduced in quarter 1 of 2023.

For opioid deaths, the trend was stable in the control provinces during the prepolicy period. In British Columbia, opioid death rates nearly doubled (from 3.1 to 6.5 per 100 000 population) between quarter 3 and quarter 4 of 2016, but declined steadily between quarter 3 of 2018 and quarter 4 of 2019. After the safer supply policy was implemented in March 2020, which also coincided with the COVID-19 outbreak, there was a spike in opioid death rates in quarter 2 of 2020, followed by a continuing upward trend in British Columbia. Although the control province also saw a spike in death rates in quarter 2 of 2020, the trend largely flattened out afterwards. After British Columbia decriminalized drug possession, the trend in opioid death rates was very similar in both British Columbia and control provinces.

Overall, the graphical analysis provides suggestive evidence of increases in opioid hospitalizations and deaths associated with the introduction of the safer supply policy and a further increase in opioid hospitalizations associated with decriminalization of drug possession. However, given the more pronounced fluctuations in British Columbia compared with the control provinces and potential challenges with the parallel trends assumption, we next present results from regression analyses using the SDID method.

### Regression Results

The base case results are presented in Table 1. The top half presents results from SDID analyses. The SDID analyses show that, compared with the prepolicy period, the safer supply period was associated with a mean increase of 1.66 opioid hospitalizations per 100 000 population (95% CI, 0.41-2.92; *P* = .009) per quarter, whereas the safer supply plus decriminalization period was associated with a mean increase of 2.94 opioid hospitalizations per 100 000 population (95% CI, 1.51-4.37; *P* < .001) per quarter. With British Columbia’s population of more than 5.6 million in quarter 4 of 2023, these increases represent absolute increases of 93 and 164 hospitalizations, respectively, or 33% and 58% relative increases, respectively, compared with 5.03 opioid hospitalizations per 100 000 population in British Columbia in quarter 4 of 2019 (before the policy was implemented). The tests that compared the coefficient estimates for the safer supply period vs the safer supply plus decriminalization period showed a significant increase of 1.27 (95% CI, 0.05-2.50) opioid hospitalizations per 100 000 population; *P* = .046). There was insufficient evidence to conclusively attribute an increase in opioid death rates to the policy changes in either the safer supply period or safer supply plus decriminalization period.

**Table 1.** Outcome Changes Associated With Safer Supply and Decriminalization Policies^a^

Variable	Safer supply period vs prepolicy period		Safer supply plus decriminalization period vs prepolicy period		*t* Test: safer supply plus decriminalization period vs safer supply period	
	Difference-in-differences estimate per 100 000 population (95% CI)	*P* value	Difference-in-differences estimate per 100 000 population (95% CI)	*P* value	Difference in coefficient estimates (95% CI)	*P* value
**Synthetic difference-in-differences**						
Opioid hospitalizations (n = 224)	1.66 (0.41-2.92)	.009	2.94 (1.51-4.37)	<.001	1.27 (0.05-2.50)	.046
Opioid deaths (n = 224)	1.06 (−3.02 to 5.15)	.61	1.59 (−2.51 to 5.69)	.45	0.53 (−0.93 to 1.98)	.48
**Traditional difference-in-differences**						
Opioid hospitalizations (n = 224)	2.26 (1.60-2.92)	<.001	3.63 (2.44-4.82)	<.001	1.37 (0.22-2.52)	.02
Opioid deaths (n = 224)	2.24 (1.34-3.14)	<.001	2.36 (1.21-3.51)	<.001	0.12 (−0.88 to 1.11)	.82

^a^ Data are from quarter 1 of 2016 to quarter 4 of 2023. Rates are numbers per 100 000 population. Estimates from synthetic difference-in-differences regressions control for proportion of children aged 0 to 17 years in the population, proportion of male participants, consumer price index, and unemployment rate in the province. Estimates from traditional difference-in-differences regressions are estimated using ordinary least squares and additionally control for province and quarter-year fixed effects.

The bottom half of Table 1 presents results from traditional difference-in-differences analyses for comparison. These analyses showed significant increases in both opioid hospitalization rates and opioid death rates during the safer supply period and safer supply plus decriminalization period.

**Figure 2.**
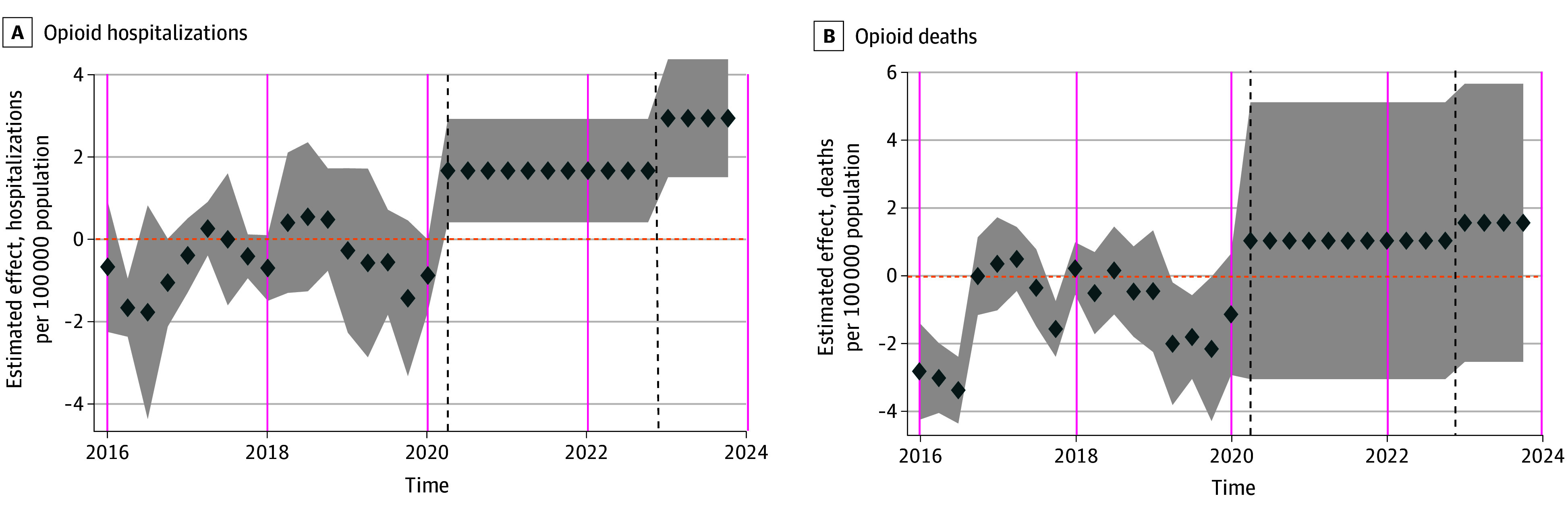
Event Study Estimates Using Synthetic Difference-in-Differences Average policy effects and their 95% CIs for the safer supply period and safer supply plus decriminalization periods are estimated using synthetic difference-in-differences. Synthetic difference-in-differences compares postpolicy difference between British Columbia and the synthetic control versus the prepolicy difference between British Columbia and synthetic control weighted by prepolicy time and control unit weights. The first vertical dashed line indicates the start of the safer supply period, and the second vertical dashed line indicates the start of the safer supply plus decriminalization period. The shaded areas indicate the 95% CIs.

Table 2 reports results from sensitivity analyses using the synthetic difference-in-differences method. Our results were robust to the exclusion of COVID-19 stringency index, naloxone policies, and economic indicators. In the regressions that excluded Ontario and New Brunswick as control provinces, the increase in opioid hospitalization rates between the safer supply period and the safer supply plus decriminalization period did not reach statistical significance but was large in magnitude. When we restricted the study period to the period after the implementation of the safer supply policy, we again found an increase in opioid hospitalizations associated with the safer supply plus decriminalization period with no evidence of increase in opioid deaths. The event study indicated no systematic differences in prepolicy trends between British Columbia and the synthetic control groups (Figure 2).

**Table 2.** Sensitivity Analyses^a^

Variable	Safer supply period vs prepolicy period		Safer supply plus decriminalization period vs prepolicy period		*t* Test: safer supply plus decriminalization period vs safer supply period	
	Difference-in-differences estimate per 100 000 population (95% CI)	*P* value	Difference-in-differences estimate per 100 000 population (95% CI)	*P* value	Difference in coefficient estimates (95% CI)	*P* value
**Excluding COVID-19 stringency index**						
Opioid hospitalizations (n = 224)	1.66 (0.48-2.84)	.006	2.94 (1.57-4.30)	<.001	1.27 (0.09-2.45)	.03
Opioid deaths (n = 224)	1.07 (−2.78 to 4.91)	.59	1.60 (−2.24 to 5.44)	.41	0.53 (−0.93 to 1.98)	.45
**Excluding naloxone policies**						
Opioid hospitalizations (n = 224)	1.82 (0.85-2.78)	<.001	3.09 (1.97-4.22)	<.001	1.27 (0.15-2.40)	.03
Opioid deaths (n = 224)	0.69 (−4.66 to 6.04)	.80	1.23 (−4.73 to 7.19)	.69	0.54 (−0.79 to 1.87)	.43
**Excluding economic controls**						
Opioid hospitalizations (n = 224)	1.93 (0.89-2.97)	<.001	3.19 (2.18-4.20)	<.001	1.27 (−0.02 to 2.55)	.05
Opioid deaths (n = 224)	1.14 (−2.94 to 5.21)	.58	1.67 (−1.88 to 5.21)	.36	0.53 (−0.81 to 1.88)	.44
**Excluding Ontario and New Brunswick**						
Opioid hospitalizations (n = 160)	1.90 (0.80-2.99)	<.001	3.06 (2.06-4.06)	<.001	1.16 (−0.39 to 2.71)	.14
Opioid deaths (n = 160)	1.30 (−3.84 to 6.45)	.62	1.80 (−3.57 to 7.18)	.51	0.50 (−1.09 to 2.09)	.55
**Study period Q2 2020 onwards**						
Opioid hospitalizations (n = 160)	NA	NA	1.56 (0.32-2.80)	.01	NA	NA
Opioid deaths (n = 160)	NA	NA	−0.02 (−1.84 to 1.79)	.98	NA	NA

Abbreviations: NA, not applicable; Q, quarter.

^a^ Data are from quarter 1 of 2016 to quarter 4 of 2023. Rates are numbers per 100 000 population. Estimates are from synthetic difference-in-difference regressions and control for proportion of children aged 0 to 17 years in the population, proportion of male participants, consumer price index, and unemployment rate in the province, province and quarter-year fixed effects.

## Discussion

This cohort study examined changes in opioid hospitalizations and deaths associated with adoption of first, the safer supply policy and then, the decriminalization policy in British Columbia relative to the control provinces. We found that the safer supply policy alone was associated with an increase in opioid hospitalization rates, which increased further after British Columbia additionally decriminalized drug possession.

The finding of an increase in opioid hospitalizations and no significant change in opioid-related deaths during the safer supply period differ from the reduction in opioid overdose deaths reported by Slaunwhite et al.^8^ This discrepancy is likely primarily due to differences in study design and focus. Slaunwhite et al^8^ assessed risk of overdose among individuals who were directly prescribed opioids via the safer supply program compared with a matched control group. Their study did not evaluate the population-level impacts, particularly on individuals who are not prescribed opioids via safer supply policies but may access them through diverted channels.

Diverted safer opioids, which have been increasingly documented, may contribute to an expanded supply of opioids in the illegal market, potentially increasing both supply and demand in unintended ways. Our study captures these broader, population-level effects, which likely explain the differing results. Some individuals prescribed safer opioids have used them as currency to buy illicit drugs,^20,21^ increasing their risk of overdose (although only 3% of opioid users have accessed safer opioids, they receive frequent supplies [for example, 14 hydromorphone pills per person/d^22^], sustaining a nontrivial and continuous supply of opioids entering the illegal market). In addition, diverted opioids could attract new users, including youth, who now have access to perceived safer opioids. Because these possible new users are opioid-naive, they face a higher risk of overdose, even from pharmaceutical-grade opioids. Moreover, these increases in demand and supply driven by safer supply opioid diversion could be further amplified in an illegal opioid market characterized by high addiction rates and spillover effects, as suggested by the thick market externalities theory.^23^ This theory posits that more users and suppliers lead to easier access to drugs, increased availability of drug-related information, and lower stigma associated with drug use, all of which attract even more participants to the illegal drug market. These spillover effects could further escalate opioid consumption and overdose incidents.

The increase in opioid hospitalizations during the safer supply plus decriminalization period compared with the safer supply period could be attributable to multiple factors. One possible explanation is that decriminalization reduced stigma associated with drug use, encouraging persons who use drugs to seek medical assistance during nonfatal overdose events. This aligns with existing evidence indicating that stigma can act as a barrier to seeking medical care. However, it is also possible that decriminalization, by reducing fear and stigma and lowering the costs of drug trading, may have inadvertently encouraged further diversion of safer supply opioids, contributing to higher opioid hospitalizations. Nevertheless, the absence of a significant increase in overdose deaths suggests that the availability of pharmaceutical-grade opioids may have mitigated some of the risks associated with unregulated opioid supply, potentially reducing the severity of outcomes.

Alongside our findings that the decriminalization policy was not associated with alleviation of the opioid crisis, there are reports that it encouraged use of drugs in public places because it disempowered the police to take action.^24^ Consequently, the government of British Columbia was prompted to recriminalize drug use in public places.^25^ There have also been concerns around safety of health care workers and a lack of appropriate housing and employment supports in the community.^24^ Although this study was unable to shed light on these social aspects, further monitoring of such unintended effects of the decriminalization policy is needed.

### Limitations

This cohort study has several limitations. First, a key goal of the decriminalization policy was to reduce disparities in drug possession arrests by race and ethnicity and other sociodemographic factors. However, we did not have data that would allow us to examine the effects of the decriminalization policy on drug-related criminal offenses or the uptake of substance use treatment. Future studies using individual record-level data are needed to examine the effects of the policy on marginalized populations. Second, because the decriminalization policy was implemented while the safer supply policy was already in effect in British Columbia, our analyses could only estimate the combined effects of both policies. Nevertheless, as supporters of decriminalization believe that its effectiveness hinges on access to a supply of safer opioids,^26^ understanding the combined effects of these policies may offer valuable insights for other jurisdictions considering similar approaches. Third, we were unable to account for the expansion of universal public insurance coverage for opioid agonist therapy. Because this expansion is expected to reduce opioid overdose hospitalizations and deaths, our estimated policy effects may be conservative. Fourth, both the SDID and the traditional difference-in-differences methods assumed that trends in outcomes in British Columbia and the control provinces would have been parallel in the absence of the policy, and that all time-varying factors have been adequately controlled for. While these assumptions are inherently untestable, the results from the event study suggest that the parallel trends assumption was unlikely to be violated. Lastly, due to limited data availability, we were only able to evaluate short-term changes in outcomes following the combined implementation of decriminalization and the safer supply policy. We were also unable to study the association of the policy with uptake of substance use disorder treatment or with hospitalizations and deaths related to nonopioid substances covered under the decriminalization policy. Further research is needed to evaluate changes in these outcomes as more data become available.

## Conclusions

In this cohort study, neither the safer opioid supply policy nor the decriminalization of drug possession seemed to alleviate the opioid crisis. On the contrary, both policies were associated with an increase in opioid overdose hospitalizations. This underscores the importance of ongoing evaluation and monitoring to fully understand the long-term effects of these policy initiatives.

## Notes

Supplement. Data Sharing Statement
