# Supplementary material for: Safer Opioid Supply, Subsequent Drug Decriminalization, and Opioid Overdoses
Source: JAMA Health Forum. 2025 Mar 21;6(3):e250101. doi: 10.1001/jamahealthforum.2025.0101 (PMC11929020; doi:10.1001/jamahealthforum.2025.0101)
Supplement: Supplement. — Data Sharing Statement [file jamahealthforum-e250101-s001.pdf]

Data Sharing Statement

Nguyen. Safer Opioid Supply, Subsequent Drug Decriminalization, and Opioid Overdoses. *JAMA Health Forum*. Published March 21, 2025. doi:10.1001/jamahealthforum.2025.0101

Data
Data available: No

Additional Information
Explanation for why data not available: This study uses publicly available data
